# The Study of Children, and Their School Training

**Published:** 1899-03

**Authors:** 


**The Study of Children, and their School Training.** By FRANCIS WARNER, M.D. Pp. xix., 264. New York: The Macmillan Company. 1898.

The excellence of Dr. Francis Warner's work in connection with the study of childhood is generally recognised; all that is here requisite is, to note the principles and construction of the book under review. Skilful adaptation of the mode of education to the needs of the particular child is the author's contention; and the proposition that all expression of the action of mind is by movement, is adopted as the basis of observation-methods. Thus, says the author, movements, balance, gestures, actions and response indicate the child's brain-state; and it should, therefore, be an object in training children to remove their faulty nerve-signs, or irregular or bad methods in movement, and with them the co-related brain-disorderliness. The earlier chapters deal concisely with the growth and development of the child's body and mind, the points to be considered in seeking indications of normal and abnormal nerve-signs; in later ones are discussed relative mental ability and psychological changes during pubescence. Subsequently comes the practical portion of the book, which deals with the removal of both motorial and mental disorders by physical training and the correction of various unhygienic conditions. Some pithy generalisations are usefully appended in the final chapter. The work should prove of interest and value to all those engaged in the education of children or in the study of them psychologically.

